# An Incremental Version of L-MVU for the Feature Extraction of MI-EEG

**DOI:** 10.1155/2019/4317078

**Published:** 2019-05-02

**Authors:** Mingai Li, Hongwei Xi, Xiaoqing Zhu

**Affiliations:** ^1^Faculty of Information Technology, Beijing University of Technology, Beijing 100124, China; ^2^Beijing Key Laboratory of Computational Intelligence and Intelligent System, Beijing 100124, China

## Abstract

Due to the nonlinear and high-dimensional characteristics of motor imagery electroencephalography (MI-EEG), it can be challenging to get high online accuracy. As a nonlinear dimension reduction method, landmark maximum variance unfolding (L-MVU) can completely retain the nonlinear features of MI-EEG. However, L-MVU still requires considerable computation costs for out-of-sample data. An incremental version of L-MVU (denoted as IL-MVU) is proposed in this paper. The low-dimensional representation of the training data is generated by L-MVU. For each out-of-sample data, its nearest neighbors will be found in the high-dimensional training samples and the corresponding reconstruction weight matrix be calculated to generate its low-dimensional representation as well. IL-MVU is further combined with the dual-tree complex wavelet transform (DTCWT), which develops a hybrid feature extraction method (named as IL-MD). IL-MVU is applied to extract the nonlinear features of the specific subband signals, which are reconstructed by DTCWT and have the obvious event-related synchronization/event-related desynchronization phenomenon. The average energy features of *α* and *β* waves are calculated simultaneously. The two types of features are fused and are evaluated by a linear discriminant analysis classifier. Based on the two public datasets with 12 subjects, extensive experiments were conducted. The average recognition accuracies of 10-fold cross-validation are 92.50% on Dataset 3b and 88.13% on Dataset 2b, and they gain at least 1.43% and 3.45% improvement, respectively, compared to existing methods. The experimental results show that IL-MD can extract more accurate features with relatively lower consumption cost, and it also has better feature visualization and self-adaptive characteristics to subjects. The *t*-test results and Kappa values suggest the proposed feature extraction method reaches statistical significance and has high consistency in classification.

## 1. Introduction

Brain-computer interface (BCI) system-based rehabilitation therapy aims to help disabled people control their injured limbs by external devices and ultimately repairs their damaged nerve pathways [1–3]. The key point of the BCI system is pattern recognition for motor imagery electroencephalography (MI-EEG) signals [4]. MI-EEG not only contains huge amounts of physiological information but also has a close correlation with the state of consciousness. Therefore, to ensure the accuracy of pattern recognition, it is very important to extract as many separable features as possible. In addition, practical application and the time consumption involved are other significant factors to consider [5].

MI-EEG is a complex non-linear time-varying and non-stationary biological signal with high-dimensional characteristics. The high dimension of the MI-EEG will raise the difficulty of feature extraction and have a further impact on the accuracy of pattern recognition. To solve the high-dimensional problems of MI-EEG, earlier researchers adopted dimension reduction methods in machine learning, such as principal component analysis (PCA), independent component analysis (ICA), and methods based on these. PCA replaced the original features with a smaller number of features. The new features were linear combinations of the old features, which maximized the sample variance and made the new features irrelevant to each other [6]. ICA, which usually involves PCA as its preprocess, is expected to decompose a signal into linear combinations of several statistically independent components [7]. These methods are easy to implement, and their weakness is obvious at the same time. The main weakness is that these methods will lose some important information due to ignoring the nonlinear characteristic of MI-EEG [8]. Manifold learning (ML) provides a better way to extract the feature of MI-EEG. ML can recover the structure of lower-dimensional manifolds from high-dimensional data and can help us to obtain the corresponding nonlinear embedded coordinates that are regarded as a meaningful representation of the reduced dimension of data [9]. According to the preserved relation between data points before and after dimension reduction, the methods of ML are divided into two types, the global approach and the local approach. The global approach is represented by isometric mapping (ISOMAP), and the local approach is represented by locally linear embedding (LLE) [10]. These two algorithms are the earliest proposed ML algorithms. They have been applied to the feature extraction of MI-EEG. Krivov and Belyaev [11] employed ISOMAP to preserve the geodesic distance of the covariance matrices to achieve dimension reduction. For the public dataset, classification accuracy is at the same level as the common spatial pattern (CSP) algorithm. Lee et al. [12] compared the effect of the feature extractions of PCA, ISOMAP, and LLE with each other and concluded that ISOMAP is better than LLE, although a lot of information is lost. From another perspective, the local approach, such as LLE, is greatly affected by the data noise, which means that, when we use the local approach to extract the nonlinear feature of MI-EEG, the data noise will affect the nonlinear structure and will further affect the classification accuracy. To overcome these limitations of ISOMAP and LLE, Weinberger and Saul [13] proposed a novel ML algorithm called maximum variance unfolding (MVU), which is based on semidefinite programming. MVU is used to maximize the Euclidean distance between data points on the premise that keep the distance in the neighborhood graph unchanging. It can detect the correct underlying dimensionality of the inputs and preserves information on both local angles and distances. In addition, Weinberger and Saul [14] emphasized that MVU is adapted to the data with noise or other particular applications by relaxing the distance-preserving constraints. However, the key step of MVU is to solve a semidefinition program, and it cannot process the huge dataset. In 2005, Weinberger et al. [15] developed an improved MVU algorithm called landmark MVU (L-MVU) to make it possible to process the huge dataset, which is based on semidefinite programming and kernel matrix factorization. Nevertheless, L-MVU also has a limitation in which we must employ the whole train data to reproduce the new low-dimension data points if we want to obtain the low-dimension data of new data points, which causes the excessive time consumption and further affects the implementation of the online application. Therefore, to overcome this shortcoming, a novel algorithm called incremental version of L-MVU (denoted as IL-MVU), which was inspired by the incremental algorithm of other ML algorithms, is presented [16–19].

However, merely extracting nonlinear features does not represent all of the information of MI-EEG. As we all know, MI-EEG has a clear time-frequency characteristic, and many earlier researchers obtained better results simply by extracting the time-frequency information. Wavelet transform (WT) was proposed to effectively obtain the time-frequency information of signals. The traditional WT is a continuous wavelet transform. However, researchers who are limited by the huge computation cost of WT usually employ the discrete wavelet transform (DWT), which is convenient for the computer calculations as it discretizes the scale and shift parameter of the continuous wavelet transform. Imran et al. [20] used DWT to extract the statistical features of MI-EEG and then employed PCA to reduce the dimension of the proposed feature vector. The k-nearest neighbor (KNN) classifier was employed to classify the features, and the average recognition accuracy was 78.26%. Even though the DWT is an efficient computational algorithm, it also suffers from a few intertwined shortcomings. For example, substantial artifacts were involved in the DWT-based reconstructed signal. The dual-tree complex wavelet transform (DTCWT), which overcame some deficiencies of the DWT, is a relatively recent enhancement of the DWT [21]. The real part and the imaginary part of DTCWT showed good information complementarity, which reduced the substantial aliasing of DWT. Minmin et al. [22] demonstrated the defect of aliasing. After that, they employed DTCWT and particle swarm optimization (PSO) to extract the feature of MI-EEG. The accuracy on the testing set reached 90%. DTCWT employs two real DWTs, which construct the real and imaginary parts of the transform and are the enhancements of DWT. Meng et al. [23] proposed a feature extraction method that combines DTCWT and the sample entropy. On the Dataset 1 of BCI Competition IV, the average classification accuracy rate of the four subjects is 87.25%. Bashar et al. [24] used DTCWT to extract the energy of coefficients as a feature from the relevant bands of motor imaginary, and the classification accuracy reached 91.07% with KNN classifier. From the aforementioned literates, we find that more researchers start to employ DTCWT to extract the time-frequency feature of MI-EEG.

In this paper, an incremental version of the L-MVU algorithm, called IL-MVU, is presented to reduce the time consumption during the testing stage, and it is combined with DTCWT, thus forming a novel hybrid feature extraction method of MI-EEG (named as IL-MD). The DTCWT is used to reconstruct the MI-EEG with every subband, and the normalization energy features of the subband signal that corresponds to the *α* wave and the *β* wave are calculated as the time-frequency feature of MI-EEG. In the meantime, IL-MVU is executed to obtain the nonlinear feature of specific subband signals with obvious event-related synchronization (ERS)/event-related desynchronization (ERD) phenomenon. Finally, we perform feature fusion for the above two types of features. IL-MD not only guarantees recognition accuracy but also meets the requirements of the online BCI system.

The remainder of the paper proceeds as follows: section 2 introduces the basic theory of the DTCWT and L-MVU algorithm. In the following section, the IL-MVU algorithm and the feature extraction method based on DTCWT and IL-MVU are introduced in detail. In section 4, the experimental steps of IL-MD are shown in details on BCI Competition 2003 Dataset 3b. The experimental results on two mentioned datasets and the discussion are shown in section 5. Finally, section 6 concludes the paper and the prospects of the future work.

## 2. Preliminary

### 2.1. Dual-Tree Complex Wavelet Transform

The decomposition of a signal with DWT will produce some frequency components that we do not expect to obtain because the low-pass and high-pass filters are not the ideal filters. In DTCWT, two real DWTs are employed to give the real and imaginary parts of the transform, and the low-pass filters of the two real DWTs should satisfy a very simple property: one should be approximately a half-sample shift of the other. In addition, DTCWT requires the first level of dual-tree filter bank (FB) to be different from the succeeding levels [25]. More details about the decomposition and reconstruction of DTCWT can be seen in Appendix.

### 2.2. Landmark Maximum Variance Unfolding

L-MVU was proposed to resolve the high time-consumption problem by choosing landmarks [15]. It uses the smaller matrix of inner products between randomly chosen landmarks to reformulate the semidefinite programming (SDP). It has already been applied to the dimension reduction [26] and the feature extraction of MI-EEG [27]. Assume that the dataset *X* ∈ *R*^*D*×*n*^ contains the high-dimensional samples *x*_*i*_(*x*_*i*_ ∈ *R*^*D*^, *i*=1,2,…, *n*), where *D* denotes the dimension of the samples and *n* is the number of the dataset *X*. The free parameters of L-MVU are the number of nearest neighbors *r* used to derive locally linear reconstructions, the number of landmarks *m*, the intrinsic dimension of the dataset *d*(*d* ≪ *D*, *d* < *m*), and the number of nearest neighbors *k* used to generate distance constraints in the SDP. Based on the parameters we set above, the steps of L-MVU are as follows.

Reconstruct each *x*_*i*_ by a weighted sum of its nearest neighbors for *r* we have set above. The reconstruction weights can be obtained by minimizing the error function:

(1) $$\begin{array}{c} \varepsilon \left(W\right)=\overset{n}{\underset{i=1}{\sum }}\left\|x_{i}-\underset{j}{\sum }W_{ij}x_{j}\right\|{}^{2}, \end{array}$$

where ∑_*j*_*W*_*ij*_=1, and *W*_*ij*_=0 if *x*_*j*_ is not the r-nearest neighbor of *x*_*i*_.

Choose first *m* sample of *X* as landmarks and compute the linear transformation *Q*. First, we define the matrix Λ=(*I*_*n*_ − *W*)^*T*^(*I*_*n*_ − *W*), and *I*_*n*_ is the *n* × *n* identity matrix. Then, partition the Λ into blocks to distinguish the *m* landmarks from other samples, as follows:

(2) $$\begin{array}{c} \Lambda =\left(\begin{array}{cc} \Lambda_{1} & \Lambda_{2}\\ \Lambda_{3} & \Lambda_{4} \end{array}\right), \end{array}$$

where Λ_1_ is the *m* × *m* submatrix of Λ and Λ_4_ is the (*n* − *m*) × (*n* − *m*) submatrix of Λ. Based on formula (10), the linear transformation *Q* was computed as follows:

(3) $$\begin{array}{c} Q=\left(\begin{array}{c} I_{m}\\ {\left(\Lambda_{3}\right)}^{-1}\Lambda_{4} \end{array}\right). \end{array}$$

Solve the SDP for the landmark kernel matrix *L* (*m* × *m*), which is the submatrix of the kernel matrix *K* in MVU. The SDP is expressed as follows.

Maximize trace (*QLQ*^*T*^) subject to

(4) $$\begin{array}{c} \text{ I}.\;{\left(QLQ^{T}\right)}_{ii}-2{\left(QLQ^{T}\right)}_{ij}\;+\;{\left(QLQ^{T}\right)}_{jj}\leq \left|\left|x_{i}-x_{j}\right|\right|{}^{2}\text{ for all }\left(i,\;j\right)\;\mathrm{with}\;\eta_{ij}=1,\\ \text{II}.\;\underset{ij}{\sum }{\left(QLQ^{T}\right)}_{ij}=0,\\ \text{III}.\;L\text{ is semidefinite,} \end{array}$$

where *η*_*ij*_={0,  1} denotes whether sample *x*_*i*_  and *x*_*j*_ is the *k*-nearest neighbor and the *k* has set earlier in this paper.

Produce the low-dimensional representation of the landmarks. First, we perform the eigendecomposition for the matrix *L* to get eigenvalues and eigenvectors. Then, the *k*^th^ element of the *i*^th^ landmarks *y*_*i*,  *k*_(*i*=1,  2,   …,  *m*) can be calculated as follows:

(5) $$\begin{array}{c} y_{i,\;k}=\sqrt{\lambda_{k}}V_{k,i}\text{,}\;k=1,\;2,\;\ldots ,\;d, \end{array}$$

where *λ*_*k*_  denotes the *k*^th^ eigenvalues of matrix *L* and *V*_*k*,*i*_ denotes the *i*^th^ element of the *k*^th^ eigenvector.

Produce the low-dimensional representation of the samples that are not selected as landmarks. These low-dimensional samples *y*_*i*_ (*i*=*m*+1,  *m*+2,   …,  *n*) are reconstructed as follows:

(6) $$\begin{array}{c} y_{i}=Q_{i}\left[y_{1}\;y_{2}\cdots y_{d}\right]. \end{array}$$

So far, we obtain the low-dimensional representation of all samples *y*_*i*_ ∈ *R* ^ *d*^ (*i*=1,2,   …,  *n*). In addition, the low-dimensional dataset is denoted as *Y* ∈ *R*^*d*×*n*^.

## 3. Methods

### 3.1. Incremental L-MVU

Inspired by the instinct that L-MVU cannot meet the time-consumption requirements when processing out-of-sample data, we proposed the incremental version of L-MVU based on its basic framework, which significantly reduces the time of the feature extraction procedure.

Assume that the dataset *X* ∈ *R*^*D*×*n*^ is the training set. The high-dimensional samples *x*_*i*_(*x*_*i*_ ∈ *R* ^ *D*^, *i*=1,2,…, *n*), which are regarded as the training sample, are contained in *X.* The free parameters *r, m, d,* and *k* are set same, as described in section 2.2. In addition, there is a new parameter *w* that denotes the number of incremental nearest neighbors. In addition, the *x*_*n*+1_ denotes the points out of the dataset *X*. Based on the above settings, IL-MVU is divided into the training and testing parts as follows.

During the training part of IL-MVU, the low-dimensional representation of *x*_*i*_(*i*=1,2,   …,  *n*), which is denoted as *y*_*i*_ ∈ *R* ^ *d*^, is produced by the L-MVU algorithm. In addition, the low-dimensional dataset is denoted as *Y* ∈ *R*^*d*×*n*^. It is worth noting that the datasets *X* and *Y* are kept in memory so that it can be used in the testing part of IL-MVU.

During the testing part of IL-MVU, the new sample *x*_*n*+1_ is put into the dataset *X* and employed its *w*-nearest neighbors to reconstruct it in the low-dimensional space. First, we find the *w*-nearest neighbors of *x*_*n*+1_ in dataset *X* and define the neighbors set as Ns. Then, we compute the incremental reconstructed weight IW by minimizing the function:

(7) $$\begin{array}{c} \varepsilon \left(\text{IW}\right)={\left|\left|x_{n+1}-\underset{j}{\sum }{\text{IW}}_{j}x_{j}\right|\right|}^{2},\;j\in \text{Ns,} \end{array}$$

where ∑_*j*_IW_*j*_=1.

Finally, the low-dimensional representation of *x*_*n*+1_ can be calculated by using the low-dimensional representation of its *w*-nearest neighbors and reconstructed weight IW, which is shown in the following:

(8) $$\begin{array}{c} y_{n+1}=\underset{j}{\sum }{\text{IW}}_{j}y_{j},\;\;j\in \text{Ns}. \end{array}$$

### 3.2. Feature Extraction Method Based on DTCWT and IL-MVU

**Figure 1:**
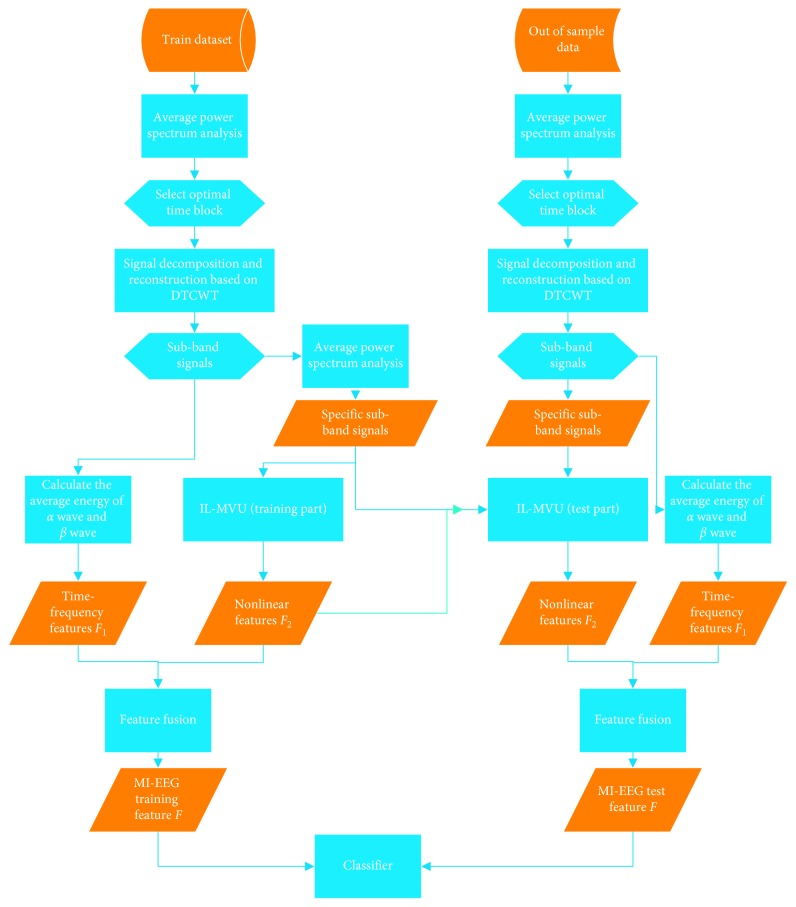
The flow chart of IL-MD.

In this section, a novel feature extraction method called IL-MD is shown in detail. The flow chart of this method is shown in Figure 1.

This method is roughly divided into five steps: signal preprocessing; signal decomposition and reconstruction based on DTCWT; average energy feature extraction; nonlinear feature extraction based on IL-MVU; and feature fusion.

#### 3.2.1. Signal Preprocessing

According to the characteristics of the MI-EEG signal, we know that the *α* wave and *β* wave include features with relatively obvious information. In addition, the ERS/ERD phenomenon is most obvious in the C3 and C4 channels [28, 29]. Therefore, according to the average power spectrum analysis of MI-EEG of the C3 and C4 channels, we can obtain the optimal time block. The average power *P*^ch^(*j*)  of the ch channel can be calculated as follows:

(9) $$\begin{array}{c} P^{\text{ch}}\left(j\right)=\frac{1}{N}\overset{N}{\underset{i=1}{\sum }}{\left[d^{\text{ch}}\left(i,j\right)\right]}^{2},\;\text{ch}=\text{c}3,\text{c}4, \end{array}$$

where *N* denotes the number of the trials and *d*^ch^(*i*, *j*) represents the *j*^th^ sample point of *i*^th^ trial in the ch channel.

Based on this, the average power spectrum of imagine left and right hands movements is drawn. In addition, the optimal time block [min, max] with the most obvious ERS/ERD phenomenon is selected according to the average power spectrum and is denoted as OT.

#### 3.2.2. Signal Decomposition and Reconstruction Based on DTCWT

As mentioned in Section 2.1 and Appendix, a signal *S*^ch^(*t*) via the J-levels DTCWT decomposition obtains its complex wavelet coefficient *d*_*j*_^ch^ and its complex scale coefficient *c*_*J*_^ch^, which are calculated in formula (19)–(26).

With the coefficients that we obtained below, we can reconstruct the signal by using formula (27). Finally, by setting the wavelet coefficients of other levels to zero, we can obtain a signal in the specific frequency band. If the sampling frequency of the signal is *f*_s_ and we perform *J*-Level DTCWT reconstruction to the signal, we will obtain the subband signals *A*_*J*_, Dt_*J*_, Dt_*J*−1_ ⋯ Dt_1_, and the corresponding frequency band ranges are [0, *f*_s_/2^*J*+1^], [*f*_s_/2^*J*+1^, *f*_s_/2^*J*^], [*f*_s_/2^*J*^, *f*_s_/2^*J*−1^],…, [*f*_s_/2^2^, *f*_s_/2].

#### 3.2.3. Calculation of Average Energy Feature

As some former researchers have demonstrated, most of the motor imagine-related information is contained in the *α* wave (8–13 Hz, namely, *μ* rhythm) and the *β* wave (13–30 Hz, namely, *β* rhythm) [28]. For general reasons, the time-frequency feature extraction of this paper is in the two mentioned waves.

First, as for the subband signals *A*_*J*_, Dt_*J*_, Dt_*J*−1_ ⋯ Dt_1_ obtained from section 3.2.2, we selected the data in the optimal time block OT, which were recorded as *S*_1_^ch^, *S*_2_^ch^,…, *S*_*L*+1_^ch^. Then, we set a sliding time window of length 2 *f*_s_. We can calculate the energy within the sliding time windows as follows:

(10) $$\begin{array}{c} E_{l}^{\text{ch}}=\overset{2f_{\text{s}}}{\underset{t=1}{\sum }}\left|S_{l}^{\text{ch}}\left(t\right)\right|{}^{2},\;l=1,2,\ldots ,J+1. \end{array}$$

After that, we choose signals whose frequency band is close to the *α* wave and the *β* wave, whose energy are recorded as *E*_*α*_^C3^, *E*_*α*_^C4^, *E*_*β*_^C3^, and *E*_*β*_^C4^. The normalization energy is computed as follows:

(11) $$\begin{array}{c} {\bar{E}}_{\text{wv}}^{\text{ch}}=\frac{E_{\text{wv}}^{\text{ch}}}{\sum_{l=1}^{L+1}E_{l}^{\text{ch}}},\;\text{wv}=\left\{\alpha ,\beta \right\}\;. \end{array}$$

The normalization energy ${\bar{E}}_{\text{wv}}^{\text{ch}}$ of two subband signals in the sliding time window is calculated by sliding one sample point at a time. From above, we can obtain four energy sequences of length (max − min − 2*f*_s_ +2), which are expressed as ES_*α*_^C3^, ES_*α*_^C4^, ES_*β*_^C3^, and ES_*β*_^C4^. Then, we obtain the ${\bar{\text{ES}}}_{\alpha }^{\text{C}3}$, ${\bar{\text{ES}}}_{\alpha }^{\text{C}4}$, ${\bar{\text{ES}}}_{\beta }^{\text{C}3}$, and ${\bar{\text{ES}}}_{\beta }^{\text{C}4}$ by calculating the average value of each energy sequence.

Finally, according to the ERS/ERD phenomenon, the average energy difference between the C3 conductor and the C4 conductor in the same wave of signal can be calculated as follows:

(12) $$\begin{array}{c} {\text{AE}}_{\text{wv}}={\bar{\text{ES}}}_{\text{wv}}^{\text{C}3}-{\bar{\text{ES}}}_{\text{wv}}^{\text{C}4},\;\text{wv}=\left\{\alpha ,\beta \right\}. \end{array}$$

As for each trial of motor imagined, we can obtain a 2-dimensional average energy feature *F*_1_, which is shown as follows:

(13) $$\begin{array}{c} F_{1}={\left[AE_{\alpha }\;AE_{\beta }\right]}^{T}\in R^{2\times 1}. \end{array}$$

#### 3.2.4. Nonlinear Feature Extraction Based on IL-MVU

As for each subband signal *S*_1_^ch^, *S*_2_^ch^,…, *S*_*L*+1_^ch^ obtained from section 3.2.3, we drew the average power spectrum, as shown in section 3.2.1. Then, we obtain S_sp_^C3^ and S_sp_^C4^, which has the most obvious ERS/ERD phenomenon in OT. To obtain the features that are more conducive to the classification, IL-MD does the following with S_sp_^C3^ and S_sp_^C4^:

(14) $$\begin{array}{c} ds\left(u,v\right)=S_{\text{sp}}^{\text{C}3}\left(u,v\right)-S_{\text{sp}}^{\text{C}4}\left(u,v\right),\;v\in \text{OT,} \end{array}$$

where *S*_sp_^ch^(*u*, *v*) denotes the *v*^th^ sample point in *u*^th^ motor imagine task of *S*_sp_^ch^. Then, we obtain the initial high-dimensional feature Tr_*u*_ ∈ *R*^(max − min+1)×1^ of *u*^*th*^ motor imagine task as follows:

(15) $$\begin{array}{c} {\text{Tr}}_{u}=\left[\mathrm{ds}\left(u,\text{min}\right),\mathrm{ds}\left(u,\text{min}+1\right),\ldots ,\mathrm{ds}\left(u,\text{max}\right)\right]\left(\right]{}^{T},\;u=1,2,\ldots ,N. \end{array}$$

After that, we construct the high-dimensional training feature set Tr ∈ *R*^(max − min+1)×*N*^ as the input of IL-MVU. Then, we set the initial parameters of IL-MVU, which is mentioned in section 3.1.

As for the samples in Tr, we obtain their *d*-dimensional feature by calculating formula (1)–(6). And for the sample out of Tr, we should execute the training part of IL-MVU first and then calculate formula (9)-(10) to obtain its *d*-dimensional feature. Therefore, for any given motor imagine task, a *d*-dimensional nonlinear feature can be obtained, and it is denoted as *F*_2_ ∈ *R*^*d*×1^.

#### 3.2.5. Feature Fusion

To make full use of the information obtained from multiple aspects, features *F*_1_ and *F*_2_ are integrated through a serial port connection. However, the order of magnitude difference between *F*_1_ and *F*_2_ is too large to affect classification. So, as to reduce the influence of the order difference on the classification, we made *F*_1_ 100 times bigger than before. Eventually, we obtain the features as follows:

(16) $$\begin{array}{c} F=\left[\begin{array}{c} F_{1}\;*\;100\\ F_{2} \end{array}\right]. \end{array}$$

To verify the effectiveness of IL-MD, the linear district analysis (LDA) classifier is selected to classify the features *F*.

## 4. Experimental Research

### 4.1. Dataset Description

To increase the persuasiveness of IL-MD, we verify IL-MD in two public datasets.

**Figure 2:**
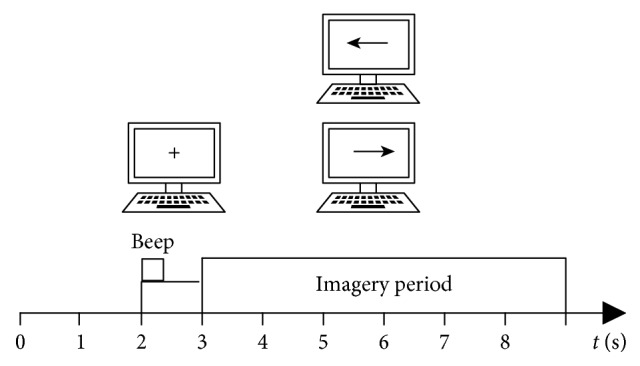
Timing schedule of Dataset 3b.

**Figure 3:**
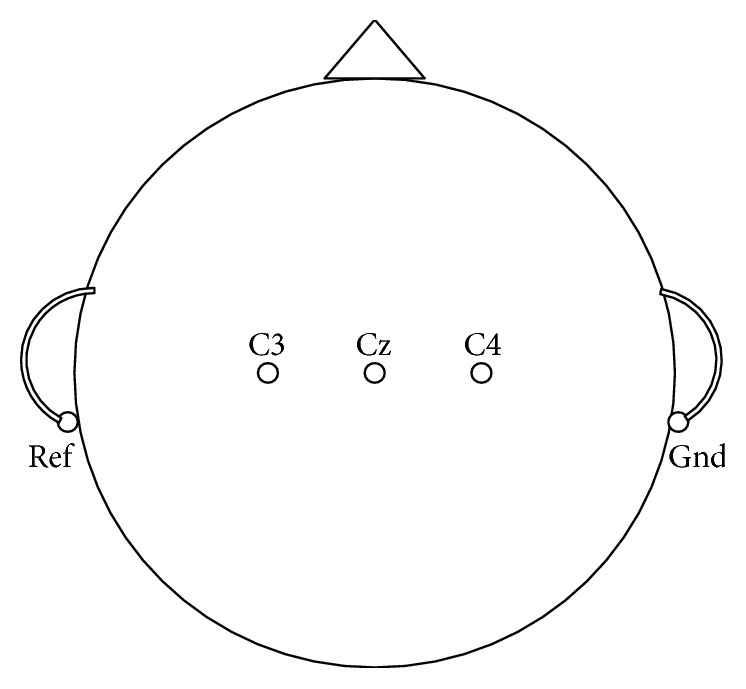
Electrode positions of C3, Cz, and C4.

The first dataset is from BCI Competition 2003 Dataset 3b provided by BCI Lab, Graz University of Technology [29], hereinafter referred to as Dataset 3b. This dataset was composed of 280 trials from 3 subjects, of which 140 were training and 140 were used to test images of left/right hand movements. The sequence diagram of each trial is shown in Figure 2. The signal was sampled at 128 Hz. The MI-EEG channels were measured over C3, Cz, and C4 conductors, using AgCl as an electrode. The electrode placement is shown in Figure 3. The placement of the electrode obeys the 10–20 electrode system.

**Figure 4:**
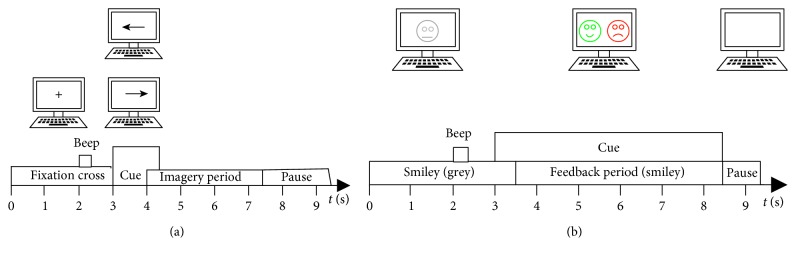
Timing scheme of Datasets 2b. (a) The first two sessions contain training data without feedback and (b) the last three sessions with smiley feedback.

The other dataset is from BCI Competition 2008 Datasets 2b provided by BCI Lab, Graz University of Technology [30], hereinafter referred to as Dataset 2b. This dataset consists of EEG data from 9 subjects, namely, B01–B09. For each subject, five sessions are provided, whereby the first two sessions were recorded without feedback, and the last three sessions were recorded with feedback. The MI tasks are same as the former dataset. For first two sessions, each session consisted of six runs with ten trials. The time schedule is shown in Figure 4(a), in which each trial has a short break of at least 1.5 seconds in the end. For the three online feedback sessions, four runs with positive feedback, denoted by a smiley symbol, were recorded (see Figure 4(b)), whereby each run consisted of twenty trials. Depending on the cue presented from 3 to 7.5 seconds, the subjects were required to move the smiley towards the left or right side by imagining left or right hands movements, respectively. During the feedback period, the smiley changed to green when moved in the correct direction; otherwise, it became red. Three channels of bipolar recording (C3, Cz, and C4) were acquired with a sampling frequency of 250 Hz. The electrode placement is the same as the former dataset, which is shown in Figure 3. The placement of the electrode also obeys the 10–20 electrode system.

In this section, the proposed feature extraction method is shown in detail using Dataset 3b. The results of the two mentioned datasets are shown in the “Result and Discussion” section.

### 4.2. Optimal Time Block Selection

The MI is a process but not a transient result. For this reason, the features of signal in different time blocks also show differences. To obtain a better result of classification, we should select the optimal time block related to the MI task based on the ERD/ERS phenomenon and apply it to the following steps.

**Figure 5:**
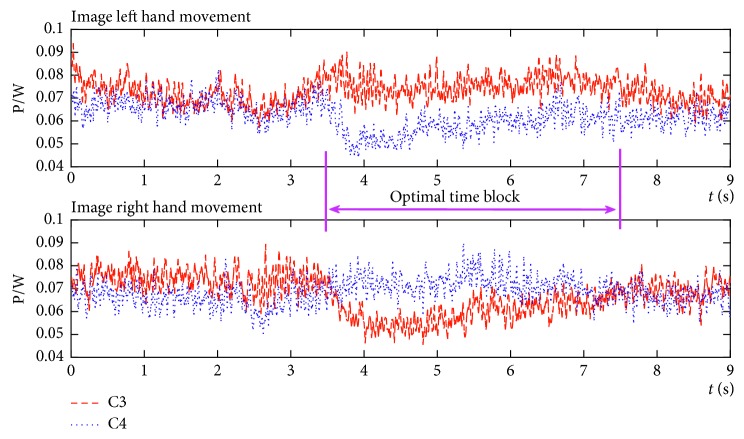
Average power spectrum of imagined left or right hand movements.

Based on the above analysis, the average power of MI-EEG over C3 and C4 conductors under two classes of MI tasks was calculated according to formula (9). The average power spectrum of the imagined left and right hands movements is shown in Figure 5.

From Figure 5, it can be seen that the time block [3.5, 7.5] *s* shows the greatest diversity under the different MI tasks. When the sample frequency of signal is 128 Hz, the sample points corresponding to this time block are denoted as approximately [450, 1000], which correspond to the optimal time block OT, which was defined in section 3.2.1.

### 4.3. Subband Selection

DTCWT can divide the frequency bands accurately, which is a recent enhancement to DWT. In addition, the MI tasks always exhibit their characteristics over a given frequency band. For this reason, IL-MD obtains each subband signal of the original signal with the decomposition and reconstruction of DTCWT. Then, the optimal frequency band will be obtained by plotting the respective average power spectrum and analyzing it based on the ERS/ERD phenomenon.

**Figure 6:**
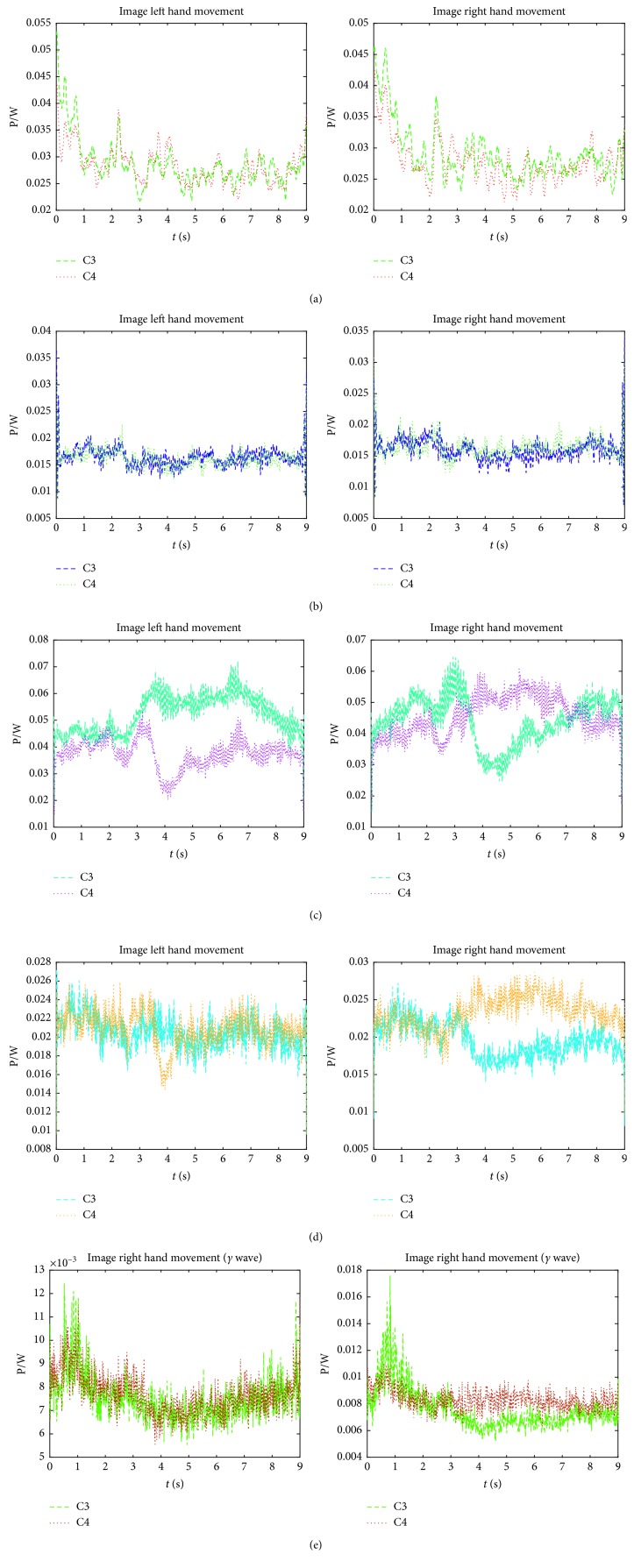
Average power spectrum of each subband signal with DTCWT-based reconstruction. Subband signal (a) *A*_4_ (*δ* wave), (b) Dt_4_ (*θ* wave), (c) Dt_3_ (*α* wave), (d) Dt_2_ (*β* wave), and (e) Dt_1_ (*γ* wave).

As mentioned in section 3.2 and in the actual situation of the dataset, the subband signal called *A*_4_, Dt_4_, Dt_3_,  Dt_2_, and Dt_1_ via 4-level DTCWT reconstruction of the original signal will be obtained, and the corresponding frequency band ranges are [0 Hz,  4 Hz], [4 Hz, 8 Hz], [8 Hz, 16 Hz], [16 Hz, 32 Hz], and [32 Hz,  64 Hz]. According to the rule of the wave division of EEG, these subband signals can correspond approximately to *δ* wave, *θ* wave, *α* wave, *β* wave, and *γ* wave. The average power spectrum is shown in Figure 6.

It can be seen in Figure 6 that the ERS/ERD phenomenon is reflected in different degrees on each subband signal. For the selected dataset, the phenomenon is barely visible in signal *A*_4_ (*δ* wave). In signal Dt_1 _(*γ* wave)  and signal Dt_4_ (*θ* wave), the phenomenon is only apparent in imaging right hand movement task. As for the rest two subband signals, Dt_2_(*β* wave) and Dt_3_(*α* wave) have the most obvious ERS/ERD phenomenon in the two classes of MI tasks, and Dt_3_(*α* wave) is even better than Dt_2_(*β* wave). So, signal Dt_3_ is selected for subsequent feature extraction on Dataset 3b. However, if the dataset is changed, the subbands selection results can be changed, namely, the multiwaves, including  *δ*, *α*, *β*,  *and*  *γ*, are all the candidates for different subjects. This reflects the subject-based characteristics of MI-EEG and will be proven in the following experiments.

**Figure 7:**
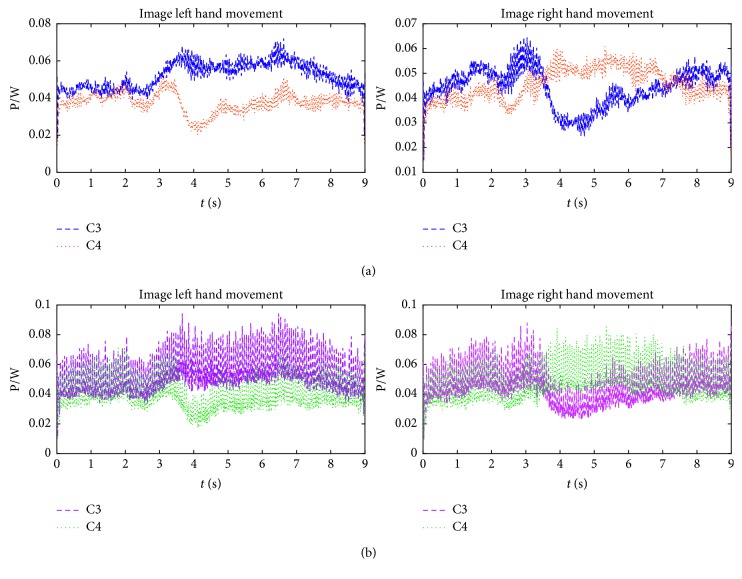
Average power spectrum of (a) Dt_3_ signal (*α* wave) reconstructed by DTCWT and (b) Dt_3_ signal (*α* wave) reconstructed by DWT.

To find the difference between the subband signals reconstructed by DWT and DTCWT, the average power spectrum of signal Dt_3_ is shown in Figure 7. It can be seen clearly from Figure 7 that the signal reconstructed by DTCWT has more obvious ERS/ERD phenomenon than that of DWT. It is because DWT may introduce substantial artifacts in signal reconstruction and cause that the corresponding ERS/ERD phenomenon is not obvious, and further more obvious features cannot be extracted. Figure 7 illustrates that DTCWT is more suitable for the subband reconstruction of MI-EEG, thanks to its perfect reconstruction characteristics.

### 4.4. Filter Bank Selection of DTCWT

The FB of DTCWT has several selectivities. And as section 2.1 was introduced, DTCWT requires that the first level of the dual-tree FB be different from the succeeding levels. In this paper, dtcwt-toolbox 4.3 was used to execute the decomposition and reconstruction of signals obtained from the previous section. This toolbox provides several FBs in the first level and in the succeeding levels of DTCWT. These FBs are shown in detail in Table 1.

Different reconstructed signals can be obtained by different combinations of these FBs. According to the classification accuracy of these reconstructed signals, the combination of the best FBs is selected for the following steps. The classification accuracy of different combinations with IL-MD is shown in Table 2. In Table 2, FB 1 denotes the FB of the first level and FB 2 denotes the FB of the succeeding levels. It can be seen that the combination of Antonini and Qshift_c obtains the highest classification accuracy. Therefore, Antonini and Qshift_c are selected as the FB in the following research.

Based on the description of section 3.2 and the selected FBs, the average energy feature can be calculated by formula (10)–(12). Finally, the 2-dimensional energy feature *F*_1_ consisting of AE_*α*_ and AE_*β*_ can be obtained and then used in the following steps.

### 4.5. Parameters Optimization of IL-MVU

To discover the spatial structure information hidden in the MI-EEG, IL-MVU algorithm is used to extract the nonlinear feature of subband Dt_3_, and CSDP 6.2.1 solver is used to solve SDP [31].

**Figure 8:**
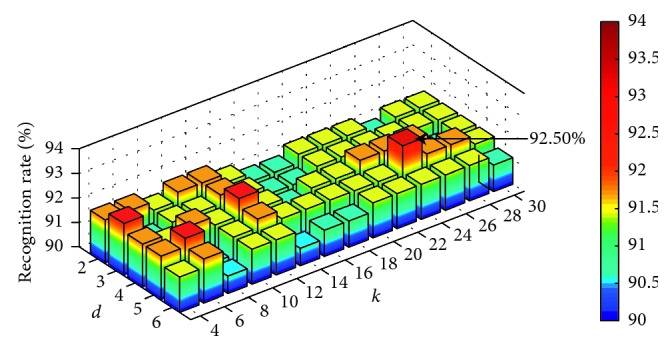
The result of parameter optimization for *d* and *k* (*r* = 52, *m* = 14).

IL-MVU algorithm has five parameters that can be adjusted. They are the number of nearest neighbors *r* used to derive locally linear reconstructions, the number of landmarks *m*, the intrinsic dimension of the dataset *d*, the number of nearest neighbors *k* used to generate distance constraints in the SDP, and the number of incremental nearest neighbors *w*. To reduce the computational expense, parameter *w* is set to 4. In fact, the experimental result shows that the parameter *w* has almost no effect on accuracy. As for the other four parameters of IL-MVU, joint optimization is performed and evaluated by the recognition accuracy. By using the traversing methods, the optimal values of these four parameters were selected.  Figure 8 illustrates the final results of parameter optimization (*r* = 52, *m* = 14). It should be noticed that the features used to classify are the combination of average energy feature *F*_1_ and nonlinear feature *F*_2_.

It can be discovered that the fluctuation range of accuracy is very small when *d* and *k* are changed. This proves that the IL-MVU algorithm has good robustness. In addition, when we set *r* to 52, *m* to 14, *d* to 5, and *k* to 24, the classification accuracy reaches its peak.

### 4.6. Feature Visualization

**Figure 9:**
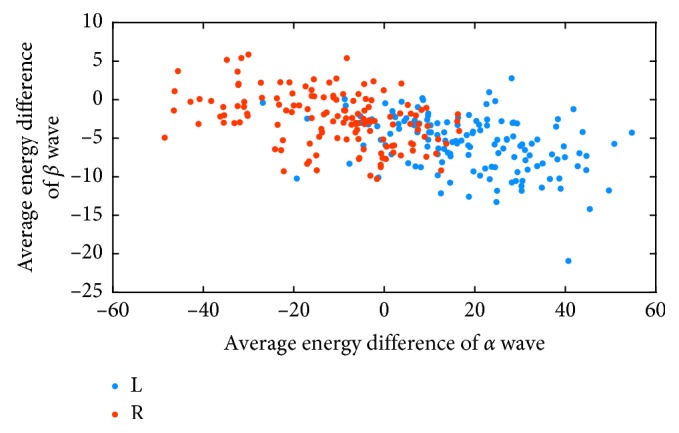
2D feature visualization about average energy feature *F*_1_.

To observe the separability of features extracted by IL-MD more intuitively, the feature visualization is carried out in this section. The feature visualization for the average energy feature *F*_1_ is shown in Figure 9. *L* and *R* in the legend denote imaging left and right hand movement, respectively.

The horizontal and vertical axes in Figure 9 stand for the average energy difference (mentioned in formula (12) of section 3.2.3) of *α* wave and *β* wave, respectively. Figure 9 illustrates that feature *F*_1_ has shown the better separability. It has a positive effect on improving the overall accuracy rate. However, the confusion of the average energy features of the two types of MI tasks still exists. It is indicated that high classification accuracy cannot be achieved by only relying on the average energy features.

**Figure 10:**
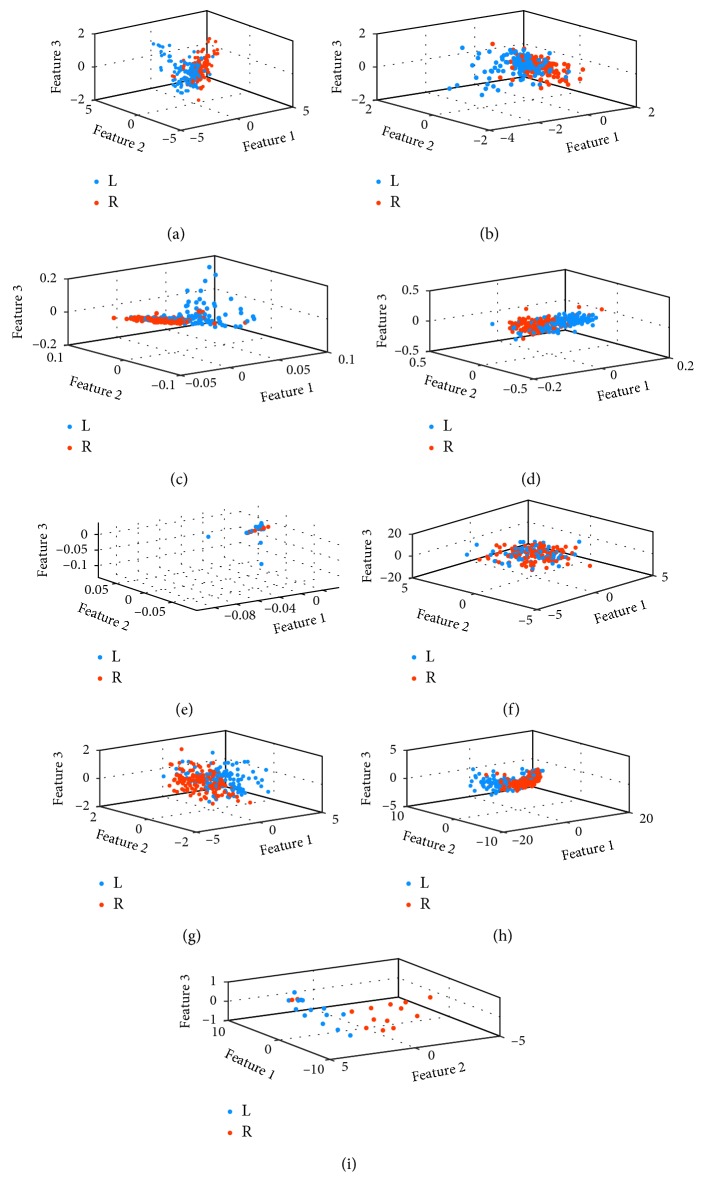
3D feature visualization about nonlinear feature *F*_2_ with common ML algorithms: (a) ISOMAP, (b) L-ISOMAP, (c) LE, (d) LLE, (e) LTSA, (f) HLLE, (g) MVU, (h) L-MVU, and (i) IL-MVU.

Furthermore, the nonlinear feature visualization shown as Figure 10 is illustrated to compare IL-MVU with the common ML algorithms. These involved algorithms are ISOMAP [32], LLE [33], Laplacian EIGENMAPS (LE) [34], Landmark ISOMAP (L-ISOMAP) [32], Local Tangent Space Alignment (LTSA) [35], Hessian LLE(HLLE) [36], MVU, and L-MVU.

The visualization results of ISOMAP, L-ISOMAP, and LE are shown as Figures 10(a)–10(c). It can be seen clearly that these three algorithms are able to classify the MI tasks that are involved in the dataset. Furthermore, L-ISOMAP is no worse than ISOMAP in visualization. In Figures 10(d)–10(f), the features that are extracted by LLE, LTSA, and HLLE are visualized. These three methods perform dimensionality reduction by retaining local information. It can be simply assumed that these three methods retain the local relation, the first derivative, and the second derivative of local relation, respectively. The visualization results of LTSA and HLLE are not as good as LLE, which proved that it is not necessary to preserve complex local relationships. Figures 10(g)–10(i) are the visualization results of MVU and its extension algorithms. A preliminary conclusion can be drawn that MVU-based algorithms have a better effect on feature visualization compared with other ML algorithms. This is because the MVU or its extension algorithm retains more useful information in reducing dimensions with its strict constraints. Moreover, compared to MVU, L-MVU has more obvious clustering distribution. As for IL-MVU, its separability is better than that of the other two methods.

## 5. Results and Discussion

### 5.1. Results and Discussion for Dataset 3b

To verify the superiority of IL-MD, a multiaspect comparison is demonstrated in this section under the same experimental conditions. The experimental environment is the Windows 10 64 bit operating system; the CPU is Intel(R) Xeon(R) E5 2683 v3; the memory is 16 GB; and software is Matlab R2017a. The 10-fold cross-validation (CV) is used in this section to reliability of experimental results. The whole 280 trials were randomly divided into 10 packages (including 28 trials per package). One package is selected as test set every time. The ten results were averaged as the final accuracy.

#### 5.1.1. Comparison of Different Classifiers

**Figure 11:**
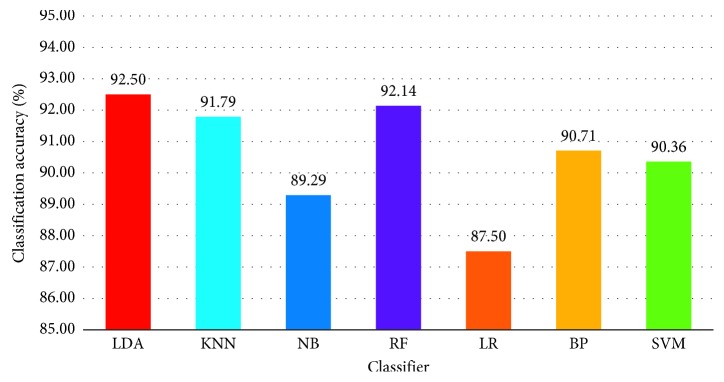
Classification results of different classifiers.

In addition to the feature extraction method, the classification accuracy is also affected by the classifier performance. In the following, IL-MD is employed to generate the hybrid features, and the commonly used classifiers, including LDA, k-nearest neighbors (KNN), naïve Bayes (NB), random forest (RF), logistic regression (LR), back-propagation net neural network (BP), and support vector machine (SVM), are applied to classify the features. The 10-fold CV experiment results are shown in Figure 11.

It is clear that the classification accuracy of LDA is higher than that of some regression classifiers, such as SVM, BP, and LR, and it also has a slight advantage over RF and KNN. The classification result of NB is worse than most of other classifiers. The classification results show that the features extracted by IL-MD do not need too complex classifiers to achieve high classification accuracy and matches best with the LDA classifier. Therefore, the LDA classifier is selected to evaluate the performance of the proposed feature extraction method.

#### 5.1.2. Comparison with Other ML Algorithms

**Figure 12:**
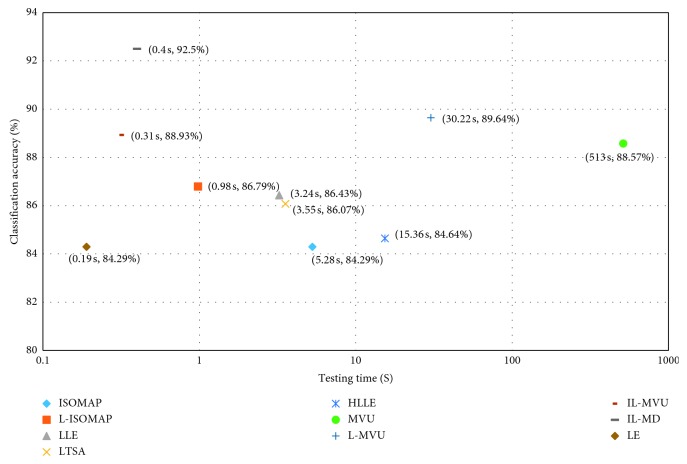
Scatter diagram of ML algorithms and IL-MD.

The proposed IL-MVU and IL-MD will be compared with the other ML methods in testing time and classification accuracy. The testing time of single sample and the 10-fold CV classification accuracy are shown in Figure 12. The parameters in the ML-based feature extraction methods have been optimized, and the classifiers are all LDA. In Figure 12, the horizontal axis denotes the single testing time of each ML method and the vertical axis denotes the recognition rates. In order to clearly demonstrate the differences in test time among various methods, the horizontal axis uses logarithmic coordinates. As we all know, an excellent feature extraction method needs to meet the requirements of high precision and low time consumption, namely, the closer to the upper left corner, the better the performance of the method.

From Figure 12, it can be seen clearly that IL-MD obtain the highest recognition rate and the lower time consumption compared with other ML algorithms. It is a benefit of the extraction of the hybrid features of MI-EEG. Furthermore, more useful information is preserved from the original data with the improved ML algorithm, i.e., IL-MVU. On the other hand, the results in Figure 12 are basically consistent with those obtained from Figure 10. Compared with ISOMAP, L-ISOMAP made great progress in recognition accuracy and time consumption. The reason is that more redundant information is discarded by choosing landmarks in the L-ISOMAP algorithm. LE obtains the lowest time consumption because this algorithm preserves less information. Therefore, the recognition accuracy of LE is the lowest too. As for the LLE-based algorithms, we can see clearly that the time consumption of LLE, LTSA, and HLLE increases successively. This is because the computation complexity of these three ML algorithms increases successively. However, the recognition accuracy reduces successively, which illustrates that LTSA and HLLE are unsuitable for the feature extraction of MI-EEG.

The MVU-based algorithms have higher recognition accuracy. Thanks to the selection of landmarks, L-MVU gains a 1% improvement compared to MVU, and the time consumption has reduced rapidly. The proposed IL-MVU algorithm, which is based on the practical application, is mapped in the out-of-sample data directly on the trained manifold according to the principle of local reservation, which greatly reduces the time consumption of feature extraction. Although the time consumption of IL-MVU is further reduced to 0.31 s, which has greatly improved the performance of online recognition for MI-EEG, it shows some decrease in the recognition rate compared with L-MVU. Therefore, it is necessary to analyze the differences of the results between the L-MVU and IL-MVU. In the following, a two-sample *t*-test is applied to identify whether there is a significant difference when they are used for MI-EEG feature extraction. The recognition rate of L-MVU and IL-MVU with 10 sets of parameters (*d*, *k*, *r*, and *m*) is used for *t*-test.

Assume that *M*_*L*_ and *M*_*IL*_ represent the mean value of the 10-fold CV's accuracy from L-MVU and IL-MVU, *S*_*L*_^2^ and *S*_*IL*_^2^ stand for the variance, and *n*_*L*_ and *n*_*IL*_ denote the number of the results. The *t*-test statistic can be calculated as follows:

(17) $$\begin{array}{c} t=\frac{M_{IL}-M_{L}}{\sqrt{\left(n_{IL}-1\right)S_{IL}^{2}+\left(n_{L}-1\right)S_{L}^{2}/\left(n_{IL}+n_{L}-2\right)\left(\left(1/n_{L}\right)+\left(1/n_{IL}\right)\right)}}. \end{array}$$

Suppose that the null hypothesis is *H*_0_, the results of IL-MVU and L-MVU come from independent random sample from normal distributions with equal means and the alternative hypothesis is *H*_1_, the results of IL-MVU and L-MVU come from populations with unequal means. The significance level was chosen as *α*=0.05. The decision rule is to reject *H*_0_, if

(18) $$\begin{array}{c} p=P\left\{t>t_{\alpha }\left(n_{IL}+n_{L}-2\right)\right\}\leq 0.05. \end{array}$$

Finally, the *p* value is equal to 0.0135, which is less than 0.05. The null hypothesis is rejected at 0.05 significance level. Therefore, the performance of IL-MVU is significantly increased compared to L-MVU. Furthermore, combined with DTCWT, IL-MD obtains the highest recognition rate of 92.50%.

#### 5.1.3. Comparison with DTCWT-Based Methods

**Figure 13:**
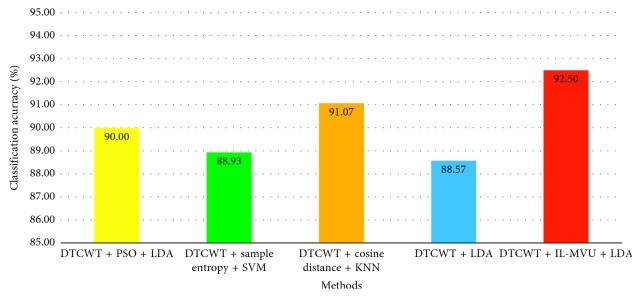
Comparison with DTCWT-based methods on Dataset 3b.

The comparison results between IL-MD and the methods of MI-EEG feature extraction based on DTCWT for the same dataset are shown in Figure 13. The feature extract method DTCWT in Figure 13 only uses the time-frequency feature mentioned in section 3. The results of the comparison methods in other literatures are not the 10-fold cross-validation result.

Figure 13 illustrates that DTCWT combined with IL-MVU obtained the highest recognition accuracy. It was proven that these two algorithms have good complementarity when applied to the feature extraction for MI-EEG. In addition, the results of the other DTCWT-based feature extraction methods are generally higher. The reason is that the frequency band can be divided more precisely by employing DTCWT, which is consistent with the frequency characteristic of MI-EEG. Take the result of Figure 12 into consideration, we find that using DTCWT or IL-MVU alone cannot reach the recognition rate above 90%. However, when these two methods are combined with the recognition rate reaches 92.50%, which is attributed to the complementarity of DTCWT and IL-MVU.

### 5.2. Result and Discussion on Dataset 2b

**Figure 14:**
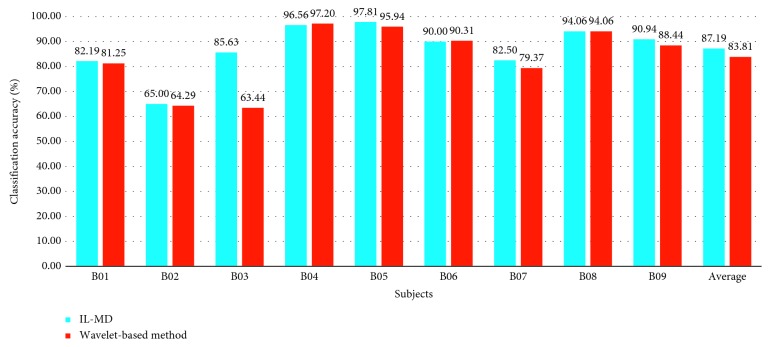
Classification rate compared with the wavelet-based method.

**Figure 15:**
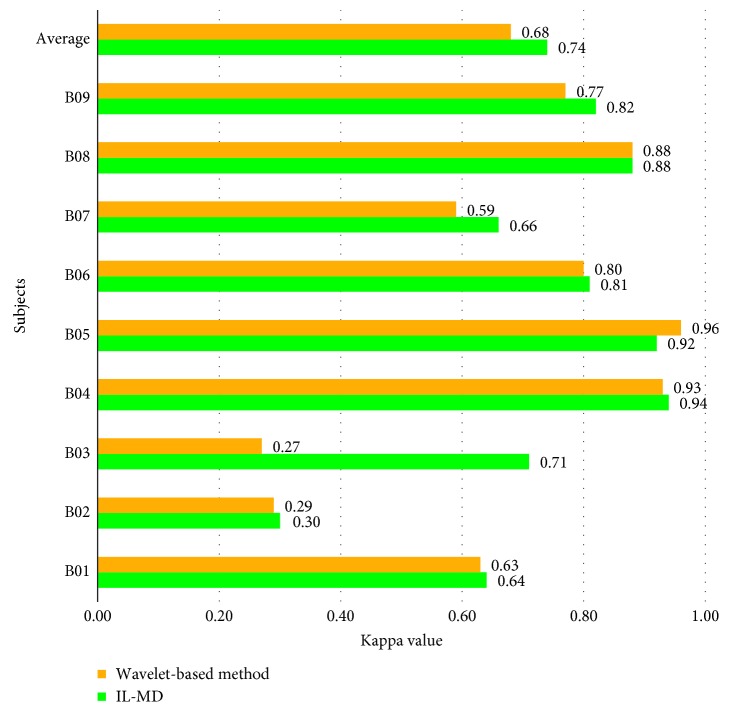
Kappa value comparison with the wavelet-based method.

To verify the self-adaptive characteristic of IL-MD, we extend this method to the multisubject dataset mentioned in section 4.1. Under the same experimental procedure as above, the subject-optimized performance is shown as Table 3. It is worth noting that the training and testing sets in this section are divided the same way as [37]. The training sets of every subject are shown in Table 3. Session 4 and session 5 of each subject are selected as their testing set. And the optimal time block is chosen from 3.5 s to 7 s for all subjects. The comparison with wavelet-based method is shown in Figures 14 and 15. The results of the wavelet-based method are from [37].

From Table 3, *α* wave and/or *β* wave are commonly selected for most of the subjects. That is because they covered the frequency band range of motor imagery. And other bands, including *δ* wave or *γ* wave, have a little correlation with MI tasks for some subjects, such as B02, B03, and B05, which demonstrates the individuation characteristics of MI-EEG.

Therefore, the individualized selection of the parameter of IL-MD according to the wave characteristics of the subjects becomes the key to improving the recognition rate of IL-MD.

From Figure 14, we can see that the average recognition rate of IL-MD is higher than the wavelet-based method. In terms of individual subjects, IL-MD obtains the better results than that of the wavelet-based method for subjects B01, B02, B03, B05, B07, and B09, it is as good as the wavelet-based method for B08, and it is slightly lower for B04. The average recognition accuracy of IL-MD makes 3.38% improvement over the wavelet-based method. In addition, the kappa values shown in Figure 15 indicate that IL-MD performs better than the wavelet-based method for most subjects. The highest improvement is obtained for subject B03, where the kappa value increased from 0.27 to 0.71. Only B05 had a slightly lower kappa value than the wavelet-based method. Although there are large variations in kappa values for different subjects, the average kappa value of IL-MD is improved by 0.06 compared to the wavelet-based method. It also indicates very good strength of class prediction and suggests that IL-MD has high consistency in classification.

IL-MD is also compared with the other methods applied to the same dataset. The experiment is finished in the same testing set, and the experiment results are shown in Table 4. The proposed feature extraction method takes an obvious advantage in the average classification accuracy and kappa value over the other methods for nine subjects. This is probably because IL-MD can excavate the individual characteristics of the subjects.

**Figure 16:**
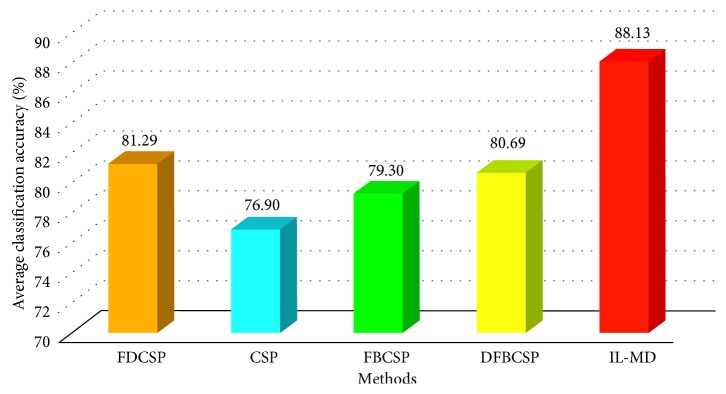
Comparison with CSP-based methods on Dataset 2b.

In addition, CSP and its extension versions have been applied in feature extraction of MI-EEG and have obtained better recognition results [43]. IL-MD was further compared with CSP-based methods, including CSP, filter bank CSP (FBCSP), discriminant filter bank CSP (DFBCSP), and frequency domain CSP (FDCSP), and the experimental results on Datasets 2b are presented in Figure 16. The experimental session is selected same as [43]. All the average recognition rates in Figure 16 are 10-fold cross-validation result.

From Figure 16, it can be seen that the improved CSP method outperforms the CSP method. However, the average recognition rate of the nine subjects using the CSP method was about 80%. The average recognition rate of IL-MD reaches 88.13%, which is superior to CSP-based methods. In order to further prove the advantage of IL-MD over CSP-based method, *t*-test over IL-MD and the best CSP-based method (FDCSP) is added. The process of *t*-test is similar with section 5.1.2. The significance level was chosen as *α*=0.05. After being calculated, the result is equal to 0.0342, which is less than 0.05. The result of *t*-test illustrates that IL-MD has significant advantages compared to FDCSP.

## 6. Conclusions

Based on the L-MVU algorithm and the principle of local reservation, an incremental version of L-MVU, i.e., IL-MVU, is proposed. It is used for nonlinear dimension reduction of specific subband signals, acquiring the subject-based nonlinear feature of MI-EEG. In addition, DTCWT is employed to extract the normalized average energy feature of subband signals corresponding to *α* wave and *β* wave. The experimental results have an advantage in feature visualization, showing that the two types of features have good separability and an obvious cluster distribution, which results in a relative higher recognition accuracy and lower time consumption. This is helpful for promoting theoretical development of manifold learning and enhancing the adaptive characteristics of feature extraction, as well. However, there is some limitation in IL-MD. One is that it cannot be applied to the multichannel signal, and the other is that it is only suited to two motor imaginary tasks. In a future study, we intend to integrate IL-MVU with a common spatial pattern (CSP) to improve its property and to develop a broad application in BCI systems. In addition, to design a more stable BCI system, the recognition of EEG generated under high pressure will also be an important aspect of our future attention [44, 45].

## Figures and Tables

**Figure 17:**
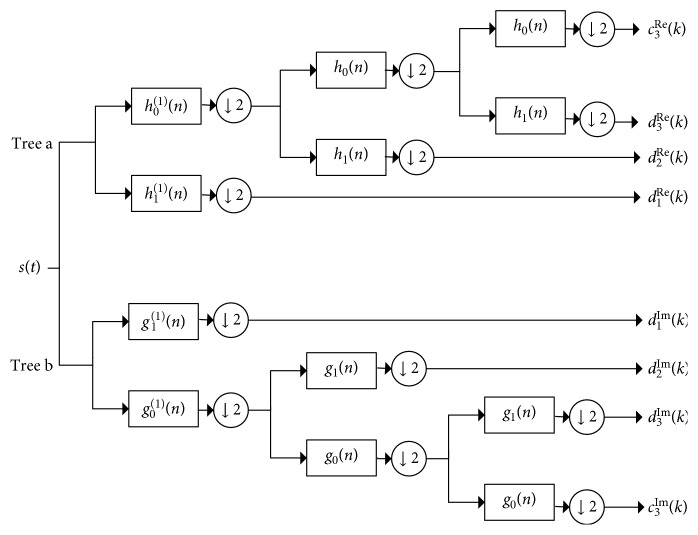
The decomposition procedures of the DTCWT.

**Table 1:** FB description of DTCWT.

FB in the first level	FB description	FB in the succeeding levels	FB description
Antonini	Antonini 9,7 tap filters	Qshift_06	Q-shift 10,10 tap filters^*∗*^
LeGall	LeGall 5,3 tap filters	Qshift_a	Qshift 10,10 tap filters^*∗∗*^
Near_Sym_a	Near-symmetric 5,7 tap filters	Qshift_b	Q-shift 14,14 tap filters
Near_Sym_b	Near-symmetric 13,19 tap filters	Qshift_c	Q-shift 16,16 tap filters
—	—	Qshift_d	Q-shift 18,18 tap filters

Note: ^*∗*^6,6 nonzero taps; ^*∗∗*^10,10 nonzero taps.

**Table 2:** Classification accuracy of different FB combinations.

FB 2	FB 1			
	Accuracy (%)			
	Antonini	LeGall	Near_Sym_a	Near_Sym_b
Qshift_06	86.07	86.07	85.71	90.00
Qshift_a	90.36	90.00	86.07	90.00
Qshift_b	80.71	81.79	80.71	92.50
Qshift_c	91.07	86.07	90.71	90.36
Qshift_d	90.36	90.36	90.71	90.36

**Table 3:** Subject-optimized performance of IL-MD.

Subjects	Parameters of IL-MVU	Training set	Wave	Recognition rate (%)
B01	*d* *=* 4, *k* = 6, *r* = 40, *m* = 13, *w* = 12	S1, S2	*α*	82.19
B02	*d* = 2, *k* = 6, *r* = 30, *m* = 6, *w* = 10	S3	*δ*	65.00
B03	*d* = 5, *k* = 4, *r* = 40, *m* = 13, *w* = 20	S2, S3	*δ*	85.63
B04	*d* = 3, *k* = 4, *r* = 40, *m* = 9, *w* = 25	S3	*α*, *β*	96.56
B05	*d* = 4, *k* = 4, *r* = 40, *m* = 9, *w* = 4	S3	*β*, *γ*	97.81
B06	*d* = 4, *k* = 26, *r* = 40, *m* = 9, *w* = 4	S1, S3	*α*, *β*	90.00
B07	*d* = 4, *k* = 12, *r* = 40, *m* = 9, *w* = 4	S1, S3	*α*	82.50
B08	*d* = 2, *k* *=* 4, *r* *=* 40, *m* *=* 11, *w* = 16	S3	*α*	90.94
B09	*d* = 3, *k* = 10, *r* = 40, *m* = 11, *w* = 8	S1, S3	*α*, *β*	87.19

**Table 4:** Comparison with other methods based on Datasets 2b.

Reference	Feature extraction	Average kappa value	Average accuracy (%)
[38]	WPD + SE-isomap	0.71	84.68
[39]	WPD + DFFS	0.70	84.06
[40]	Improved DFBCSP	0.63	81.02
[41]	KL-divergence + CSP	0.62	77.22
[42]	CNN + SAE	0.61	75.10
This paper	IL-MD	**0.74**	**87.19**

## Data Availability

In this manuscript, previously reported two datasets were used to support this study and are available at http://bbci.de/competition/ii/ and http://www.bbci.de/competition/iii. These datasets are cited at relevant places within the text as references [29, 30].

## Appendix

### Decomposition and Reconstruction of DTCWT

Figure 17 shows the decomposition procedures of the DTCWT. As Figure 17 shows, the real component tree is represented by Tree a and the imaginary component tree is represented by Tree b.

The wavelet coefficients and scale coefficients in Tree a and Tree b can be shown as follows:

(19) $$\begin{array}{c} d_{j}^{\text{Re}}\left(n\right)=2^{j/2}\int_{-\infty }^{\infty }s\left(t\right)\Psi_{h}\left(2^{j}t-n\right)dt, \end{array}$$

(20) $$\begin{array}{c} d_{j}^{\text{lm}}\left(n\right)=2^{j/2}\int_{-\infty }^{\infty }s\left(t\right)\Psi_{g}\left(2^{j}t-n\right)dt, \end{array}$$

(21) $$\begin{array}{c} d_{J}^{\text{Re}}\left(n\right)=2^{j/2}\int_{-\infty }^{\infty }s\left(t\right)\Phi_{h}\left(2^{j}t-n\right)dt, \end{array}$$

(22) $$\begin{array}{c} d_{J}^{\text{lm}}\left(n\right)=2^{j/2}\int_{-\infty }^{\infty }s\left(t\right)\Phi_{g}\left(2^{j}t-n\right)dt, \end{array}$$

where *j* is the scale factor and *j* *=* 1, *…*, *J*, *n* is the sample number, and *s*(*t*) is the signal. Ψ_*h*_, Ψ_*g*_,  Φ_*h*_ , and Φ_*g*_ are the functions of the wavelet transform, which can be expressed by dual-tree FBs, such as *h*_1_(*n*), *g*_1_(*n*), *h*_0_(*n*), and *g*_0_(*n*).

Afterward, the complex wavelet coefficient *d*_*j*_^ *C*^(*n*) and the complex scaling coefficient *c*_*J*_^*C*^(*n*) of the dual-tree complex wavelet, which formed by its real and imaginary parts as formulas (19)∼(22), can be obtained by the following equations:

(23) $$\begin{array}{c} d_{j}^{\left(C\right)}\left(n\right)=d_{j}^{\text{Re}}\left(n\right)+id_{j}^{\text{Im}}\left(n\right), \end{array}$$

(24) $$\begin{array}{c} c_{J}^{\left(C\right)}\left(n\right)=c_{J}^{\text{Re}}\left(n\right)+ic_{J}^{\text{Im}}\left(n\right). \end{array}$$

The reconstruction procedure of the DTCWT is simple. The wavelet coefficients and the scaling coefficients could be inverted as

(25) $$\begin{array}{c} d_{j}\left(t\right)=2^{\left(j-1\right)/2}\left[\overset{\infty }{\underset{n=-\infty }{\sum }}d_{j}^{\text{Re}}\left(n\right)\Psi_{h}\left(2^{j}t-n\right)+\overset{\infty }{\underset{k=-\infty }{\sum }}d_{j}^{\text{Im}}\left(n\right)\Psi_{g}\left(2^{j}t-k\right)\right], \end{array}$$

(26) $$\begin{array}{c} c_{J}\left(t\right)=2^{\left(j-1\right)/2}\left[\overset{\infty }{\underset{n=-\infty }{\sum }}c_{C}^{\text{Re}}\left(n\right)\emptyset_{H}\left(2^{J}t-n\right)+\overset{\infty }{\underset{k=-\infty }{\sum }}c_{J}^{\text{Im}}\left(n\right)\Phi_{g}\left(2^{J}t-k\right)\right]. \end{array}$$

And the signal *s*(*t*) could be expressed as

(27) $$\begin{array}{c} s\left(t\right)=\overset{J}{\underset{j=1}{\sum }}d_{j}\left(t\right)+c_{J}\left(t\right). \end{array}$$

Note that we can obtain a signal in each subband easily by setting the wavelet coefficients of other subbands to zero. When we process the nonstationary signal with a time-frequency characteristic, such as MI-EEG, we could employ DTCWT to obtain the expected frequency bands accurately and to extract their time-frequency feature.
